# Development and validation of an impedance biosensor for point-of-care detection of vascular cell adhesion molecule-1 toward lupus diagnostics

**DOI:** 10.4155/fsoa-2017-0047

**Published:** 2017-07-07

**Authors:** Anjan Panneer Selvam, Andi Wangzhou, Michael Jacobs, Tianfu Wu, Chandra Mohan, Shalini Prasad

**Affiliations:** 1 Department of Bioengineering, University of Texas at Dallas, Richardson, TX 75080, USA; 2 Department of Internal Medicine, University of Texas at Southwestern Medical Center, Dallas, TX 75390, USA; 3 Department of Biomedical Engineering, University of Houston, Houston, TX 77204, USA; 4 Cognition and Neuroscience, School of Behavioral and Brain Sciences, University of Texas at Dallas, Richardson, TX 75080, USA

**Keywords:** impedance biosensor, rapid urine screening test, VCAM-1

## Abstract

**Aim:**

Systemic lupus erythematosus is an autoimmune disease that requires chronic monitoring. In this study, we demonstrate a proof-of-concept study of a highly attractive noninvasive strategy for monitoring systemic lupus erythematosus through biomarker quantification.

**Results:**

This sensor technology requires 50 μl of urine to detect and quantify vascular cell adhesion molecule-1 in 15 min. The sensor used nonfaradaic detection to demonstrate performance with and without detection antibody. Binding of immunoassay and target biomarkers were quantified with an impedance electrical immunoassay and correlated with an equivalent circuit.

**Conclusion:**

The novel sensor technology demonstrates detection in the range of 8 fg/ml to 800 pg/ml and comparative analysis with ELISA platforms was performed for 12 patient urine samples.

## Lay abstract

A proof-of-concept portable biosensing system for rapid screening of systemic lupus erythematosus through the use of a biomarker has been demonstrated. This sensor is an electronic urine test, which screens for vascular cell adhesion molecule. The efficacy of the sensor has been validated using a small cohort of patient urine samples that have been evaluated using standard clinical methods. This biosensor is the first demonstration of a consumer-friendly point-of-care test suitable for home monitoring.

Point-of-care (POC) biosensors are portable devices that can be used to detect and quantify biomarkers in the diagnosis and prognostic analysis of various diseases [1,2]. With the increase in knowledge of the factors that can affect internal body functions, there is a high demand for screening and validating various biomarkers before a decision on diagnosis is made. POC biosensors can be used in the clinical setting with patients presenting for testing or as companion monitors for at-home diagnostics. Presymptomatic diagnosis has paved the way toward better treatment strategies and disease-tracking approaches, which have consequentially increased the demand for POC biosensors that have high sensitivity and specificity for assaying physiological fluids [3,4]. Additionally, POCs are highly attractive for monitoring autoimmune diseases.

Under normal conditions, the immune system detects pathogens, including but not limited to viruses and bacteria, which invade the human body. The immune system detects these foreign bodies through surface or expressed biomarkers. Antibodies released by the immune system then engage with moieties on these pathogens. The immune system also detects cells infected by pathogens and lyses the cell or activates the cell's self-lysis program to avoid pathogen releases [5]. Systemic lupus erythematosus (SLE), commonly called lupus, is a systemic autoimmune disease in which the immune system functions abnormally and recognizes healthy cells as pathogens. This causes the immune system to attack and lyse one's own healthy cells. SLE is a potentially serious form of autoimmune disease, which affects multiple organs including heart, lungs, liver, kidneys, blood vessels, joints, nervous system and skin [6–9].

The incidence of SLE is approximately 1.0 to 8.7 per 100,000, and shows a rising trend [10,11]. The prevalence also varies with gender and ethnicity. The survival rates after 5, 10 and 15 years of SLE diagnosis are 96, 93 and 76%, respectively [10]. Despite the progress made in recent years, the pathogenesis of SLE still remains unclear [12]. While there is still no cure for SLE, the current treatment for SLE is immune-suppression, which could lead to adverse side effects [13]. The complex diagnosis of SLE relies on multiple criteria and is an area of growing scientific research [14,15]. Recorded statistical data indicate the rate of possible symptoms of SLE occurrence to vary greatly from 3 to 95% [16], which may lead to false positive results for SLE diagnosis, and also late diagnosis. A major diagnostic approach in SLE is based on detection of biomarkers, such as antinuclear antibodies [17]. Newer biomarkers, such as those selected from global urinary or serum protein scans, can be detected in patients’ body fluids, prior to the appearance of symptoms [17]. This raises hope for earlier diagnosis and management of renal involvement in this disease [18].

There are a number of emerging biomarkers that can potentially be used as indicators of renal disease in SLE. Human vascular cell adhesion molecule-1 (VCAM-1) has been recently identified as a promising urinary biomarker candidate. VCAM-1 is a protein that is encoded by the VCAM-1 gene and a member of the immunoglobin superfamily. VCAM-1 provides support for tethering and adhesion of leukocytes to endothelial cells and also acts as a ligand for integrins [19]. A study by Shui *et al*. has shown that the expression of VCAM-1 is regulated by tumor necrosis factor and IL-1 [20]. Additionally, the expression of VCAM-1 has been shown to be increased in the mesangial cells of mice as presented in Shui *et al*. [21]. In another clinical study, human urinary VCAM-1 levels were correlated to higher renal activity, hematuria, proteinuria and pyuria [22]. Higher expression was also corroborated to studies mentioned by Ballardie *et al*., which show significantly higher levels of VCAM-1 in patients with lupus nephritis versus normal or control populations [23]. This calls for a need in developing a portable and at-home usable VCAM-1 urine test, which can enable rapid and noninvasive diagnostic and prognostic monitoring to better understand its role in the disease.

Currently, sandwich immunoassays are predominantly used for detecting VCAM-1 protein concentrations [24]. A major limitation of this mode of diagnosis is the complexity in the implementation of the assay. With all the reagents prepared, the incubation time of ELISA plates takes about 2 days, and with the pre-incubated plate, the actual test time takes longer about 5 h. Hence, there is a significant need for alternative methods that are rapid, reliable and ultrasensitive. Electrochemical biosensors are promising tools for POC testing due to low cost, ease of miniaturization and possibility of integration with multi-array tools [25,26]. The use of electrochemical impedance spectroscopy in biological applications has been reported since as early as 1925 [22]. Electrochemical Iimpedance spectroscopy (EIS)-based biosensing has been used for protein biomarker detection from a number if sensor substrates with both simple and complex buffers [27–29]. This paper demonstrates the design, development and testing of a cost effective and robust, simple to use biosensor, which can profile VCAM-1 levels using electrochemical impedance spectroscopy (EIS). In this work, we report for the first time an EIS-based sensor platform for the detection of VCAM-1. We use gold microelectrodes with chemically conjugated immunoassays to detect and quantify VCAM-1 in buffer solutions and human urine samples to demonstrate the potential of EIS in POC applications. We present a calibration response curve and a semi-quantitative analysis of VCAM-1 detection on the developed sensor platform and compare it with measurements from ELISA platforms.

## Materials & methods

### Sensor fabrication

**Figure 1.**
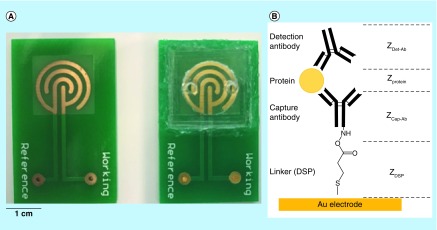
**Electrical biosensor platform for rapid and robust detection of vascular cell adhesion molecule-1.** **(A)** Optical micrograph showing the gold microelectrodes and encapsulant **(B)** schematic of sandwich immunoassay for VCAM-1 detection using DSP as the linker for conjugation with gold electrode surface. DSP: Dithiobis succinimidyl propionate; VCAM: Vascular cell adhesion molecule.

Figure 1 shows the sensor platform used in this work. The sensor consists of two components: a printed circuit FR-4 board with gold microelectrodes electroplated on it and a microfluidic sample chamber fabricated with polydimethlysiloxane (PDMS). Electrical contacts were interfaced with a lead-free solder to connect the microelectrodes with a bench-top potentiostat for electrical and electrochemical characterization. The microfluidic sample chamber was designed to hold a sample of 100 μl. The design and dimensions were chosen to enable rapid sample diffusion upon addition to the inlet port. The sensor assembly (microelectrode + microfluidic sample chamber) was bonded with heat-curable silicone to ensure a liquid-proof seal. The microfluidic channels were flushed with isopropyl alcohol and 0.15 M phosphate-buffered saline (PBS; BP2944100, Thermo Fisher, IL, USA) followed by vacuum desiccation prior to introduction of the immunoassay reagents.

Impedance spectroscopy and cyclic voltammetry was used to characterize electrical performance of the fabricated sensor system and identify the electrical nature of molecules used in each step of the immunoassay.

Dithiobis succinimidyl propionate (DSP) (Thermo Scientific, MA, USA) was used to cross-link the capture antibody on the sensing electrodes. DSP has two functional ends: one with a disulfide that binds to the gold electrode and another with National Health Service (NHS) ester for binding with a capture antibody moiety. The electrodes were treated with 10 mM DSP dissolved in DMSO for 1 h to create a thiol linkage to the gold electrodes and leaving an open NHS ester for immobilizing capture antibodies. A capture antibody specific to VCAM-1 was used for the detection of VCAM-1 in buffer solutions and urine. The electrodes were incubated with the capture antibody solution for 30 min. Post-antibody immobilization, the sensing electrodes were treated with 1× Superblock (Thermo Scientific) blocking buffer solution to hydrolyze unbound NHS ester groups. Detection of VCAM-1 antigen was monitored with and without the use of a specific VCAM-1 detection antibody. Immunoassay reagents were used from DuoSet ELISA for Human VCAM-1/CD106 (#DY809, RnD Systems, MN, USA).

### Electrochemical impedance spectroscopy for characterization of binding events on sensor platform

We use electrochemical impedance spectroscopy to characterize the binding of the different entities on the electrode surface as they create a coulombic potential, which could be screened as impedance changes. We used a modified Randle's circuit to fit the impedance spectra and identify the electrochemical nature of binding leading to classification of specific versus nonspecific interactions on the microelectrodes. Electrical double layer capacitance is reflective of the columbic potentials developed due to biomolecular binding. Experimental parameters used in this study included V_rms_ = 10 mV and frequency range of 10 Hz to 10 kHz.

### Calibration of sensor platform for detection of VCAM-1

Prior to testing with patient urine samples, the sensor was first calibrated by testing with VCAM-1 calibration standards in PBS buffer.

The target antigen was calibrated for signal changes occurring due to: binding of VCAM-1 with capture antibody probes on the sensing electrodes and binding of detection antibody to VCAM-1 – capture antibody conjugate on the sensing electrodes. The calibration response was performed for a total of n = 5 replicates and error was calculated as the SD over mean. Curve fitting was performed to establish a correlation between varying concentrations of the calibration standards with respect to impedance measured. As a negative control, each of the sensor arms was also tested for nonspecific signal with the blank buffer solution and urine from healthy volunteers.

### Detection of VCAM-1 from human urine samples

The sensor platform was tested for feasibility and accuracy of detection of VCAM-1 in human urine to show proof-of-feasibility for detection using impedance spectroscopy toward application in companion diagnostics. A total of 12 samples were tested and a semi-quantitative analysis was used to compare with results obtained from ELISA. The samples were diluted 5000× in a diluent buffer to match detection range of the designed sensor platform. The diluent buffer solution was composed of 10 mM PBS at pH 7.4 with 150 mM NaCl and 0.1% sodium azide. All patient urine samples were obtained, de-identified and used in accordance with an institution-approved protocol from UT Southwestern Medical Center, Dallas, TX, USA. The patient samples after dilution were added at a volume of 100 μl to the sensor. The sample was incubated for 15 min. Impedance measurements were performed without the need for any wash steps.

## Results

### Baseline electrical characterization of sensor surface

**Figure 2.**
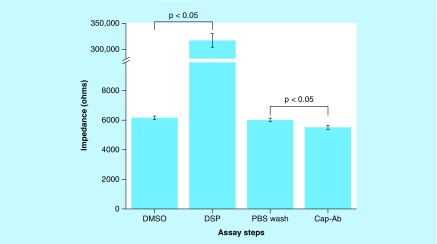
**Validation study to assess linker binding and capture antibody immobilization.** Impedance change indicates immobilization of resistive DSP linker molecule. PBS wash was performed prior to addition of antibody. Impedance decrease indicates covalent immobilization of capture antibody on electrode surface. Statistical significance threshold was set at 0.05. Error bars represent SD over mean for n = 3 replicates. DSP: Dithiobis succinimidyl propionate; PBS: Phosphate-buffered saline.

The sensor platform was first characterized for baseline impedance with blank 0.15 M PBS solution, urine and DMSO. These measurements were used as a reference to validate cross-linking and immunoassay implementation. DSP cross-linker dissolved in DMSO at 10 mM concentration was incubated on the sensor for 30 min to achieve complete and effective functionalization. Impedance measurements were recorded and a maximum change in signal was identified at 10 Hz. Figure 2 shows the trend of impedance changes measured for DMSO (control – 6 kΩ) followed by increase in impedance for DSP conjugation (305 kΩ). The data were collected and plotted for a total of n = 6 sensors, and 3× DMSO washes were performed on the sensor and impedance changes were less than 5% with respect to measurements at DSP cross-linking. A 3× PBS was next performed to wash off any DMSO or unbound DSP molecules. Anti-VCAM-1 capture antibody was added to the sensing electrode and incubated at room temperature for 30 min. Impedance measurements were carried out before and after capture antibody treatment to identify success in immobilization. Figure 2 shows the impedance changes observed at a frequency of 10 Hz pre- and post-antibody treatment. The decrease in impedance from the PBS assay step to the addition of antibodies corresponded to the immobilization of the capture antibody. The conjugation of capture antibody to the NHS ester on the DSP linker results in the formation of a defined electrical double layer, which is further characterized in the following section. To validate the immobilization, the sensor surface was washed three-times with PBS and impedance measurements were repeated. There were no statistically significant differences between the three washes. The antibody coated sensor was vacuum desiccated and stored at 2–8°C and showed stability up to 3 days without any additional packaging.

### Noise signal estimation

**Figure 3.**
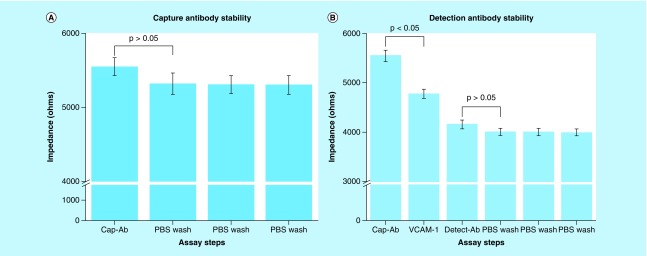
**Stability of antibody molecules and noise signal estimation.** **(A)** Capture antibody immobilization followed by 3× PBS wash. **(B)** Detection antibody binding to VCAM-1 followed by 3× PBS wash to demonstrate stability. Noise signal estimated as change in impedance from immobilization steps for both conditions. Statistical significance threshold was set at 0.05. Error bars represent SD over mean for n = 3 replicates. PBS: Phosphate-buffered saline; VCAM: Vascular cell adhesion molecule.

To estimate the signal due to background buffer and nonspecific binding, we performed a noise signal classification study using PBS. A total of n = 5 sensors were immobilized with the capture antibody of interest and incubated with the blank buffer for 30 min. Impedance measurements were carried out and the change in impedance for noise signal was calculated as the difference (ΔZ_noise_) obtained by subtracting the impedance obtained at PBS wash after Superblock (control) from the impedance measured PBS. The impedance measured at the control assay step (PBS wash after Superblock) and for PBS is shown in Figure 3.

To characterize the noise signal due to nonspecific binding by the detection antibody, we performed a study where the sensor was first saturated with capture antibody, followed by a 3× wash with PBS. Detection antibody was then added to the sensor to measure the impedance, and the change from the previous PBS wash was used to estimate the noise signal (ΔZ_noise-detect_).

### Identifying the impedance–concentration relation for VCAM-1 biomarker

A serial dilution of each of the purified target protein of interest, VCAM-1, was prepared in PBS over the range of 8 fg/ml to 800 pg/ml. The aliquots were prepared individually and used for the calibration response study to identify the analytical parameters of the sensor device. The capture antibody saturated sensor surface was treated with Superblock buffer to seal-off any unbound linker sites. A PBS wash was performed following this, and the impedance measurements were recorded. The impedance measurement at this step was defined as the baseline/control value (ΔZ_Baseline_). The impedance measurements were analyzed at a frequency of 10 Hz as it showed maximum change in signal amplitude with successive steps of binding in the electrical double layer.

**Figure 4.**
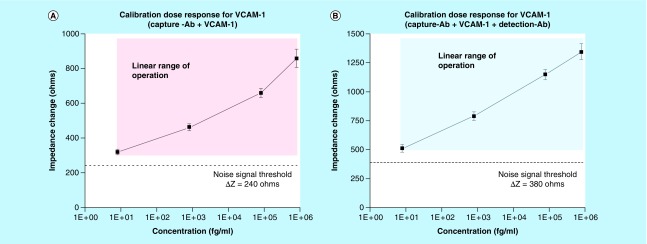
**Calibration dose response correlating concentration of vascular cell adhesion molecule-1 in the range of 8 fg/ml to 800 pg/ml with change in impedance observed at 10 Hz.** **(A)** CDR for VCAM-1 detection without detection antibody and **(B)** CDR for VCAM-1 with detection antibody added. Error bars represent SD over mean for n = 5 replicates. CDR: Calibration dose response; VCAM: Vascular cell adhesion molecule.

All impedance changes for the subsequent testing with the target biomarker dilutions were calculated with reference to the baseline/control impedance. Figure 4A & B shows the calibration response for VCAM-1 over the concentration range of 8 fg/ml to 800 pg/ml. Error bars represent SD over mean for a total of n = 5 replicates. The experiments in this section were performed to calibrate the sensor for binding between the capture antibodies and target VCAM-1 proteins only.

The sensor platform showed a linear range of impedance change between 320 and 900 Ω over the concentration range of 8 fg/ml to 80 pg/ml. At doses <8 fg/ml of VCAM-1, the change observed was lesser than the noise signal threshold (ΔZ_noise_) and the measured signal could not be classified as a distinguishable change due to binding events. The limit of detection for the sensor platform was defined as 8 fg/ml. For concentrations >800 pg/ml, the impedance signal showed saturation and hence was defined as the upper limit of detection for VCAM-1.

We performed another study to understand the impact of using detection antibodies in a nonfaradaic electrochemical sensor platform. Detection antibody at a concentration of 75 ng/ml was used to transduce the signal from VCAM-1 binding to the capture antibody. In this case, the sensor platform showed a linear range of impedance change between 800 and 1400 Ω for the concentration range of 8 fg/ml to 800 pg/ml. The measurements were taken after adding detection antibody followed by a PBS wash to reduce any noise signal due to electrostatically bound moieties. The change observed for the target concentration range was above the noise signal threshold, demonstrating confidence in the signal observed with VCAM-1 binding.

### Electrical circuit model for estimating impedance changes at electrode–electrolyte interface

**Figure 5.**
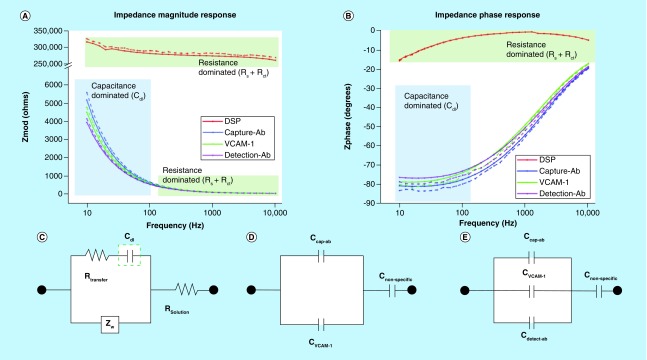
**Electrical circuit model for impedance estimated at the electrode–electrolyte interface.** Impedance spectra for **(A)** magnitude and **(B)** phase for different steps in immunoassay implementation. Straight lines represent experimental data and dashed lines represent simulated data. **(C)** Equivalent circuit model for analyzing sensor platform impedance response, C_dl_ is the double layer capacitance, R_sol_ is solution resistance, R_ct_ is charge transfer resistance and Z_w_ is Warburg impedance. Expanded C_dl_ parameter for immunoassay **(D)** without and **(E)** with detection antibody is presented.

The goal of using an electrochemical detection technique is to transduce changes occurring due to antibody-antigen binding in a rapid and robust manner. The interaction of a fluid with ionic constituents (PBS or urine in this case) with the electrode surface results in a charge distribution region, which is perturbed with any subsequent functionalization as a part of the capture immunoassay. We modeled the impedance spectrum of the sensor platform using the equivalent electronic circuit model shown in Figure 5C and used MATLAB to determine the electrical nature of the individual and lumped circuit components. Figure 5A & B shows the Bode plot comparing the theoretical and simulated Zmod values and Zphase values for the assay at four key steps – DSP functionalization, capture antibody conjugation, target VCAM-1 and detection antibody binding. For this modeling and analysis, we picked the highest concentration of VCAM-1 in the observed linear range for this sensor platform, which is 80 pg/ml. The solid lines in the plot correspond to experimentally obtained values and the dotted lines correspond to theoretically estimated values. The experimental data when compared with the simulated results with statistical analysis showed no significant difference between the experimental and theoretical values, at a threshold of 5%.

The observed Bode plot for Zmod versus frequency indicated that the sensor platform for steps of DSP conjugation showed a highly resistive behavior with Zmod not varying over the frequency range of 1 Hz to 10 kHz. This can be attributed to the high electrical resistive nature of DSP. We used a modified Randle's circuit to study the contribution of each element as shown in Figure 5C. The observed behavior indicates a lumped resistance domination with conjugated DSP transducing charges as a charge transfer resistance (R_ct_) and excess DSP in solution as solution resistance (R_s_). Following this, conjugation of the capture antibody and further assay steps of VCAM-1 binding and detection antibody binding resulted in an exponential behavior at low frequencies indicative of capacitance dominated operation. The observed plot for Zphase versus frequency showed a negative value of phase corresponding to ‘lag behavior’ as seen for a capacitor element in an electrical/electronic circuit. The system showed maximum negative phase at a frequency of 10 Hz. This confirmed the formation of an electrical double layer with the conjugation of the capture antibody and further accumulation or perturbation of charges in the capacitive electrical double layer upon VCAM-1 binding and detection antibody binding. In the immunoassays where no detection antibody was added, we modeled the capacitance behavior at low frequencies where maximum signal change occurs, as shown in Figure 5D. With any immunoassay, on microelectrodes, we have a percentage of nonspecific binding from the reagent molecules and we modeled that as C_nonspecific_ to account for any electrostatically adsorbed antibody or target VCAM-1 proteins on the electrode surface. We also performed simulations on a model that accounted for the additional binding of the detection antibody. The equivalent circuit is shown in Figure 5E. The simulations demonstrated the accuracy of the model toward further testing of more complex sample matrices.

### Detection of VCAM-1 in human urine samples

**Figure 6.**
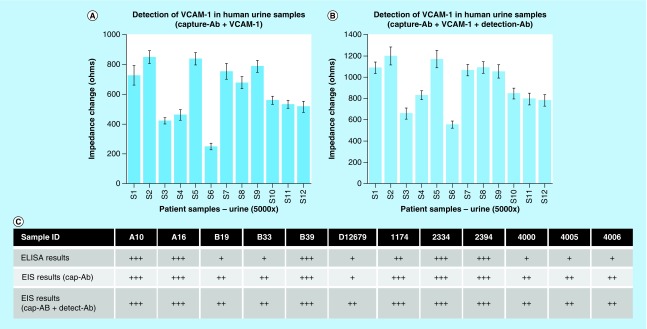
**Assessment of VCAM-1 in human urine samples.** Impedance change response for VCAM-1 biomarker levels in patient urine samples **(A)** without and **(B)** with detection antibody added, and tested on the sensor platform. **(C)** Semi-quantitative analysis was performed to compare ELISA results with sensor platform responses. Marker representation levels: 500 (+), 500–1500 (++) and >1500 μg/ml (+++). Error bars represent SD over mean for n = 3 replicates. VCAM: Vascular cell adhesion molecule.

A blinded cohort of 12 patient urine samples were tested on the sensor platform and the equivalent ELISA test, in order to evaluate the performance of the biosensor. The original patient urine samples were tested by ELISA for quantifying the concentration of VCAM-1 and to use as a comparator. The sensor platform presented here required a 5000-fold dilution of the urine samples in order to detect VCAM-1 in the concentration range shown in Figure 4. The sensors used in this study were fabricated as described above for the calibration experiments with VCAM-1 capture antibody on the sensing electrodes. Sensing was performed for VCAM-1 with and without the secondarily added detection antibody. The impedance signal response observed for the 12 samples are shown in Figure 6A & B. In this preliminary study, we performed a semi-qualitative analysis to demonstrate the ability of the sensor platform to classify VCAM-1 concentrations as high, medium or low. Values of +, ++ and +++ were assigned to the sensor platform output based on VCAM-1 concentrations of 500, 500–1500 and >1500 μg/ml respectively. Figure 6 shows the comparison of the ELISA results with the sensor platform, with and without adding the detection antibody.

## Discussion

The presented nonredox electrochemical sensing technology provides a novel, rapid, simple and inexpensive biomarker detection method that directly transduces binding of VCAM-1 to capture antibodies with or without the help of a detection antibody. This technology utilized the immobilization of capture antibodies on gold microelectrodes resulting in the formation of a charged electrical double layer. Biomolecules and chemical entities are generally charged species, which form counter-ions in the presence of a charged electrolyte. The conjugation of biomolecules and cross-linkers on the gold microelectrodes results in the formation of structured electrical double layer with charge distribution varying relative to the immunoassay being used. The binding of target biomarkers to the specific antibodies results in highly specific capacitance changes, transduced as impedance and electrical phase changes that are specific to double layer perturbations. The binding of nonspecific molecules does not cause the signal changes at specific low frequencies of interest and this provides a versatile modality for quantifying concentration of bound target protein. To enhance specificity, we also tested the additional contribution of a second (detection) antibody, and observed an improvement to the overall signal range, thus enhancing platform operation.

We performed initial studies with blank solutions (free from VCAM-1) to ensure stability of the antibody and to characterize the baseline noise signals. The impedance spectroscopy results revealed that the maximum impedance magnitude shifts occurred at low frequencies of 10–100 Hz. Thus, the bulk conductance of the solution is screened out. The impedance changes at the low frequencies are dominated by double layer capacitance changes due to perturbations with conjugated and bound moieties. We performed detection of VCAM-1 in the dynamic range of 8 fg/ml to 800 pg/ml, with and without the secondary addition of detection antibodies, and observed linear operation and signal response in both cases. Further electrical system analysis was performed with the help of two newly designed equivalent circuit models (for with and without addition of detection antibody) to understand the response of the sensor platform and to characterize the nature of each contributing reagent. We have previously studied the importance of understanding the electrical nature of similar immunoassay components toward better interpretation of impedance response [30]. The immunoassay implemented on the microelectrodes formed a stable electrical double layer, which was perturbed with the binding of each immunoassay reagent and the target VCAM-1 protein as well. These changes were reflected in the capacitance dominated impedance element – Zimaginary. The correlation for varying concentrations was established in the calibration dose response. The accuracy of the designed electrical model was confirmed with a variation of less than 5% from the experimental results obtained.

In order to demonstrate the applicability of the technique for POC and companion diagnostics, we performed validation studies with ELISA to detect VCAM-1 in a total of 12 lupus nephritis patient urine samples. A limitation we observed in this study was the varying dynamic range of detection for existing techniques as well as the electrical assay presented in this manuscript. To match the range of detection, we had to perform a 100× dilution for the ELISA-based assay and a 5000× dilution for the novel sensor platform. We observed variability in performance for a few patient samples and attribute this to the precision of the equivalent circuit model. In the case of urine, nonspecific molecules can contribute as both resistance and capacitance elements. The addition of electrical elements to account for the nature of urine can improve the precision of the sensor. Alternative strategies, which utilize a simple wash step with microfluidics, may also improve the accuracy of detection. In addition, increased sample sizes may also help to reduce the impact of subject-to-subject variance in these studies. The device shows stability in performance and with adequate packaging can easily have a shelf life of greater than 1 year. In conclusion, the preliminary results demonstrate efficacy of the sensor technology for use in POC setting toward quantification of various biomarkers in urine, which can improve diagnosis and prognosis of SLE.

## Future perspective

Current technical approaches for identifying and diagnosing diseases and their primary cause depends heavily on optical immunoassays and demand a laboratory setting and experienced personnel to undertake these tests. Rapid screening POC tests require robustness in performance to work directly with body fluids in a simple and easy manner. Implementing electrical transduction modalities in a nonfaradaic manner can potentially open exciting avenues in creating practical POC and companion diagnostic tools for various diseases. We expect that these results would create a positive impact and pave the path toward using electrochemical/electrical impedance spectroscopy to perform sensitive detection for reliable clinical diagnosis. This technique can be easily extended to assay various physiological fluids for a multitude of biomarkers, toward rapid and robust POC screening. Finally, significantly enhanced sensing and selectivity can be potentially attainable by using custom electrical impedance models for different application types. Automated microfluidic systems can be easily coupled to enhance high-throughput screening and quantification of biomarkers in a multiplexed format.

**Summary points**

**Background**

- Rapid and robust screening for biomarkers in diseases such as systemic lupus erythematosus and nephritis can enable early diagnosis and reliable disease monitoring.
- Impedance biosensors have shown robustness in detecting target biomarkers in a nonfaradaic method, thus enabling ease of use and implementation.

**Experiment**

- Sandwich immunoassay designed for ELISA was used with thiol-conjugated gold microelectrodes to develop an electrical immunoassay.
- Electrical circuit model for analyzing impedance response was developed and validated with simulations.
- Validation with human urine samples from patients was performed to compare device performance to current gold standard.

**Results & discussion**

- Noise signal levels for capture and detection antibody with buffer solutions were characterized.
- Calibration dose response correlating impedance changes with vascular cell adhesion molecule-1 concentrations was performed.
- Equivalent circuit model to analyze and characterize impedance response is presented to identify contributions of each immunoassay entity.
- Demonstration of vascular cell adhesion molecule-1 detection in human urine samples and validation with ELISA in a semi-quantitative fashion is presented.

**Conclusion**

- The developed biosensor shows preliminary efficacy for developing point-of-care device and can be extended for use with various biomarkers in complex body fluids.
